# Congenital cystohepatic duct found during laparoscopic cholecystectomy

**DOI:** 10.1093/jscr/rjae401

**Published:** 2024-06-04

**Authors:** Svetlana Shumarova, Anton Koichev, Manol Sokolov, Angel Arabadzhiev, Vesela Karamisheva

**Affiliations:** Department of Surgery, University Hospital “Aleksandrovska” Sofia, Bulgaria, Medical University, 1 Georgi Sofijski Blvd, 1431 Sofia, Bulgaria; Department of Surgery, University Hospital “Aleksandrovska” Sofia, Bulgaria, Medical University, 1 Georgi Sofijski Blvd, 1431 Sofia, Bulgaria; Department of Surgery, University Hospital “Aleksandrovska” Sofia, Bulgaria, Medical University, 1 Georgi Sofijski Blvd, 1431 Sofia, Bulgaria; Department of Surgery, University Hospital “Aleksandrovska” Sofia, Bulgaria, Medical University, 1 Georgi Sofijski Blvd, 1431 Sofia, Bulgaria; Department of Obstetrics and Gynecology, Second Gynecology Clinic SBALAG “Maichin dom”, Sofia, Bulgaria, Medical University, 1431 Sofia, Bulgaria

**Keywords:** cystohepatic duct, cholecystectomy, laparoscopic

## Abstract

This case shows the need for in-depth knowledge also on congenital biliary anomalies that can become subject to iatrogenic damage. The patient is 44-years old with echographically proven cholelithiasis with complaints of intermittent pain in the right upper quadrant. During laparoscopic cholecystectomy, after identification of cystic duct and cystic artery, after their clipping and resection and subsequent mobilization of the gallbladder from the liver parenchyma, a bile duct was opened. Subsequent identification revealed a cystohepatic duct, which is a rare anatomic anomaly. Plastic surgery was performed on the tangential lesion of the right hepatic duct and placement of a transcistic drain, as well as a drain from the right hepatic duct through the Fateri papilla. Postoperative transdrainage cholangiography established the integrity of the bile ducts and the free passage of contrast to the duodenum.

Intraoperative identification of only two structures entering the gallbladder during cholecystectomy—cystic duct and cystic artery—is mandatory.

## Introduction

Any change in the anatomy of the gallbladder and extrahepatic bile ducts, as well as the lack of preoperative preparation, can lead to intraoperative iatrogenic damage, which in turn can cause postoperative complications. The most common cause of biliary tract damage is pericholecystic changes occurring after severe inflammatory in this area, but anatomical varieties and congenital biliary anomalies, which although rare, can seriously complicate the overall intraoperative picture should not be underestimated. Patel *et al.* [1] describe 12 common congenital anomalies, including the presence of cystohepatic duct, occurring in 1%–2% of cases. We report a case of congenital cystohepatic duct that was found intraoperatively after laparoscopic cholecystectomy.

## Case presentation

We report a case of a 44-year-old women who presented to the emergency department with 1 week of abdominal pain in the right upper quadrant and epigastrium with nausea and vomiting unresponsive to conservative therapy. The patient mentions a similar pain 5 months ago, when cholelithiasis was diagnosed and the refused surgical treatment for personal reasons. On presentation, she had normal vital signs. The physical examination showed tenderness in the right upper quadrant of the abdomen without signs of peritoneal irritation and a negative Murphy’s sign. The blood parameters are within normal limits. Abdominal ultrasound examination demonstrated hydrops of the gallbladder with multiple calculi of different size in the lumen, thickened walls, no evidence of pericholecystic collection, and changes in the bile duct.

**Figure 1:**
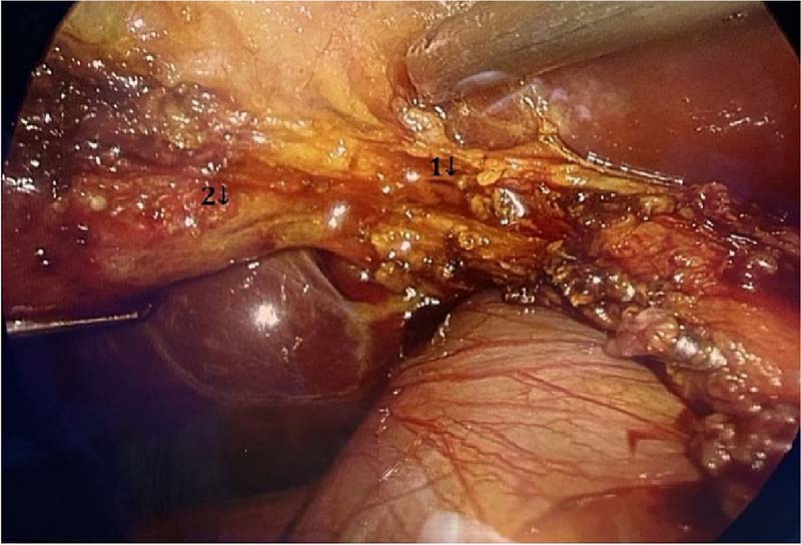
Visualization of the biliary lesion; (1) tangential damage to the right hepatic duct; (2) gallbladder with lateral traction.

**Figure 2:**
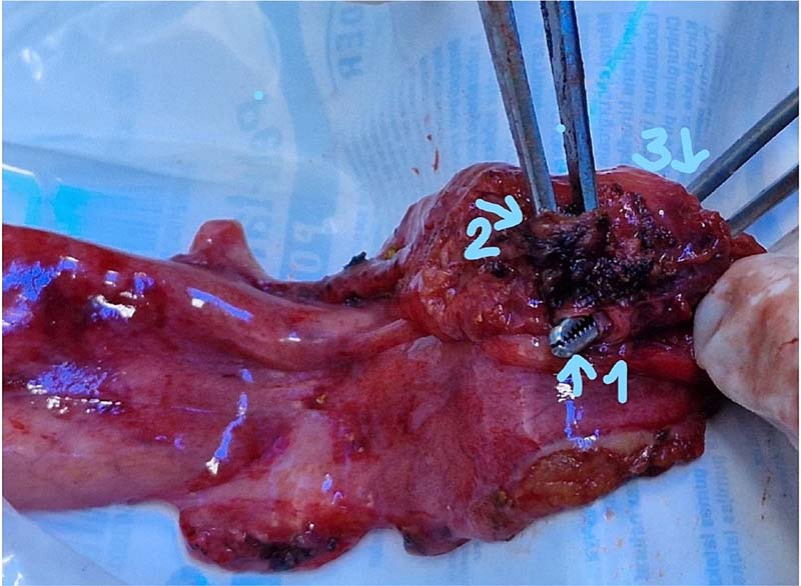
Gallbladder (1) distal part ot the cystic duct; (2) the part connecting with the right hepatic duct; (3) entrance to cystic duct from the lumen of the gallbladder.

**Figure 3:**
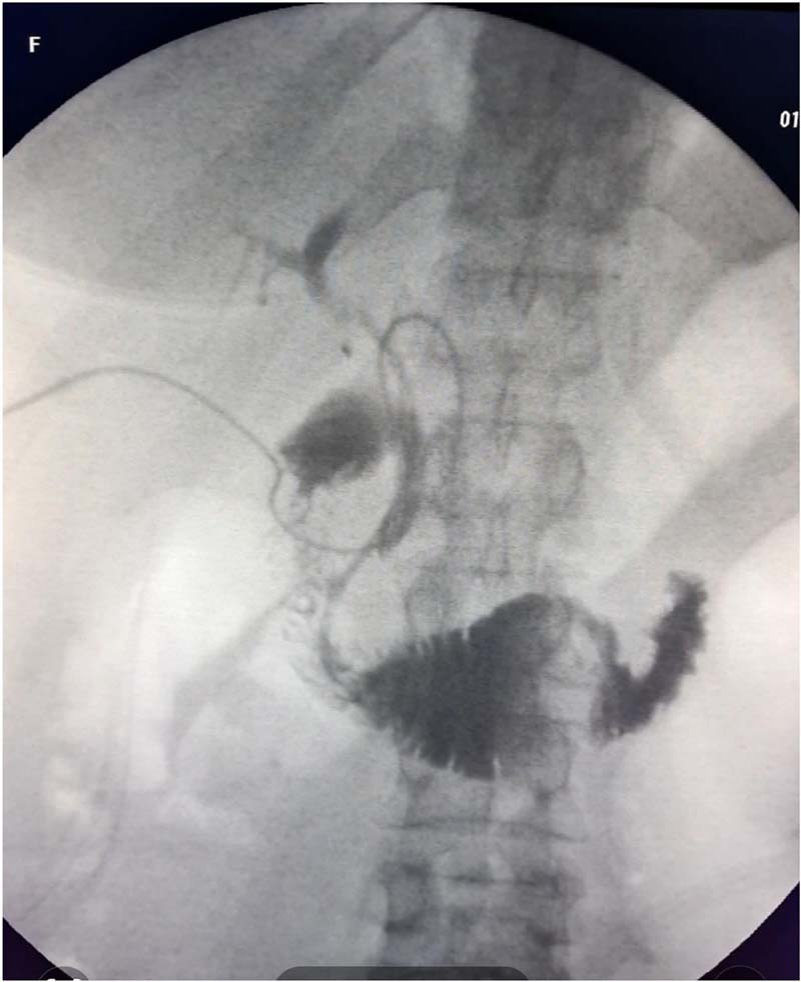
Postoperative transdrainage cholangiography.

The patient was scheduled for a laparoscopic cholecystectomy the following day. Intraoperatively, an enlarged gallbladder hydrops with the presence of various caliber calculi in the lumen, the largest of which measured 2 cm/d, was found. The contents of the bladder are punctured and evacuated to facilitate its grasping in the fundus area and creating a good exposure of Callot’s triangle. Using a four trocar technique, a dissection was performed in the triangle of Callot, noting the difficult manipulation in the area between cystic duct and common hepatic duct. The cystic duct is identified to its confluence with the common bile duct at a 45° angle. The cystic artery was also visualized, after which clips were placed on both structures after ensuring that they entered the gallbladder. Dissection of the gallbladder in its distal part, attached to the liver parenchyma above Rouvier’s line, was started using electrocautery and hook. The tissue density of a limited area in the distal part between the wall of the gallbladder and the liver parenchyma was dissected using a hook. A bile duct was opened in its unusual anatomical location (Fig. 1). Due to lack of equipment for intraoperative cholangiography, it was converted to open access and subsequent revision of the extrahepatic bile ducts. The common bile duct and right–left hepatic duct were mobilized to the site of entry into the liver parenchyma. A parietal lesion of the right hepatic duct just above the confluence and anatomical integrity of the common bile duct and left hepatic duct were found. After examining the removed gallbladder, it was found that it was immediately flowing into a duct which connects the cystic duct with the right hepatic duct as depicted in Fig. 2. A plasty of the right hepatic duct was performed, and prior to this duodenotomy and papillotomy were performed by mobilization of the duodenum according to Kocher. A protective drain was placed in the choledochus draining from the right hepatic duct to the ampulla of Vater. Separetely, the clips previously placed on the cystic duct were removed and a transcystic drain was placed; a leak test was performed at the plastic site of the right hepatic duct. The postoperative period was without complications. From the transcystic drain, the secretion of bile stopped on the fourth postoperative day. On the eighth postoperative day, transdrainage cholangiography was performed; the common bile duct and bought hepatic ducts were imaged, and free passage of the contrast material through the duodenum was established (Fig. 3). The transcystic draine was removed on the 20th day of surgery.

## Discussion

The normal anatomic location of the cystic duct is its entry into the common hepatic duct on its right lateral site. A frequent debate among surgeons is how to avoid iatrogenic damage to the biliary tree during cholecystectomy. What are the most common causes of such an injury? Should preoperative imaging be mandatory in every patients undergoing elective cholecystectomy?

Of utmost importance is the identification of the common bile duct and cystic duct during cholecystectomy to avoid lesions and subsequent postoperative complications. Given that cholecystectomy is the most common digestive tract surgery, the debate about iatrogenic damage to the bile ducts during cholecystectomy is completely understandable and the controversy regarding iatrogenic damage to the bile ducts during cholecystectomy reported from 0% to 0.6% by Hussain *et al.* [2]. Other authors reported a significantly higher percentage—15% of biliary tract injury, based on 68 cholecystectomies performed [3]. Based on 120 performed endoscopic retrograde cholangiopancreaticography and/or magnetic resonance cholangiopancreaticography, they prove that iatrogenic injures are higher in abnormal anatomy (*P* = .04) [3]. They also report abnormal anatomical variations in 30% of cases. They are part of the risks factors for difficult laparoscopic cholecystectomy describe by Hussain *et al.* [2], as well as male gender, advanced age chronic cholecystitis with a thickened wall, short, and wide cystic duct, biliodigestive fistula, obesity.

Anatomical varieties include congenital anomalies of the bile ducts, and in 2013, Patil *et al.* [1] described a total of 12 cases of anomalies of cystic duct, of which 11 were congenital. They should be well known by the surgeon given the difficulties they can cause during cholecystectomy. The length and course of cystic duct and its connection pattern with common hepatic duct (CHD) are variable and it is not necessary to outline its entire course and its point of confluence with CHD during cholecystectomy according to Gupta *et al.* [4], as it would place the bile duct at risk of injury, especially in cases where the cystic duct adheres to CHD due to inflammation. The first difficulty in performing a dissection in the region of Calot’s triangle during cholecystectomy in the case described by us is precisely such an increase in the angle between cystic duct and CHD, as illustrated in Fig. 1. All the time on the cholecystectomy but actually a mobilization of the structures cephalade relating to the defensive line described by Gupta *et al.* [4] as R4U line (Rouviere’s sulcus-Segment 4-Umbilical fissure).

Another important point according to Gupta *et al.* [4] is the visualization of the lower part of the cystic plate—a flat ovoid fibrous sheet, which is an anatomical part of the systemic lining of the liver [5]. According to Strasberg *et al.* [5], a layer of loose areolar tissue between the gallbladder wall and cystic plate thickens in the hilus region. This was the critical place for us during the laparoscopic cholecystectomy, where the proximal part of hepatocystic duct we incorrectly interpreted it as the distal part of cystic plate due to tight adhesion to the wall of the gallbladder. To avoid leakage of bile during or after surgery, it is necessary to mobilize the space between this thickening and the surface of the liver, even at the risk of bleeding, then to clip it. In this way, we would have avoided a conversation to an open operation and subsequent plastic surgery of right hepatic duct. Another criticism we would make to ourselves, and a recommendation to all surgeons, is the performance of intraoperative cholangiography at the slightest suspicion of difficult differentiation of structures during surgeon. In this case, the lack of such equipment at the time of the operation justifies us.

After a detailed systematic review of the literature by Eikermann *et al.* [6] and the question of whether it is possible IOC to prevent bile duct injury, they find that its use is controversial and cannot be routinely recommended. However the panel, they say, agrees that IOC allows for early identification of bile duct injury as long as it correctly interpreted.

Given the radiation load that can bring to each patient and the impossibility of supplying such equipment in all treatment units, on the other hand, it makes it difficult to think in the direction of routine use. The most reliable method for accurate preoperative diagnosis is MRI cholangiography, as it can predict associated pathological changes [8]. A disadvantage of this method is the financing part, which is why hardly every surgical unit would afford to be included in the algorithm for mandatory preoperative diagnosis of the hepatobiliary system.

## Conclusion

Intraoperative identification of only two tubular structures entering the gallbladder during cholecystectomy—cystic duct and cystic artery—is mandatory. In case of any suspicion of additional structures beyond those indicated intraoperative cholangiography is recommended.
